# Anaphylaxis in atypical cold urticaria: case report and review of literature

**DOI:** 10.1186/s13052-018-0578-6

**Published:** 2018-11-13

**Authors:** Elisa Benelli, Giorgio Longo, Egidio Barbi, Irene Berti

**Affiliations:** 1 Department of Medical, Surgical and Health Sciences, University of Trieste, Via dell’Istria, 65, 34137 Trieste, Italy; 2 Institute for Maternal and Child Health IRCCS “Burlo Garofolo”, 34137 Trieste, Italy

**Keywords:** Anaphylaxis, Cold urticaria, Cold stimulation test, Atypical cold urticaria

## Abstract

**Background:**

Cold-induced urticaria is a kind of physical urticaria characterized by the appearance of wheals after exposure to cold. The atypical form is a rare sub-type characterized by appearance of hives even in areas not directly exposed to the cold and by a negative cold stimulation test. Its diagnosis is often challenging because of the lack of specific tests and it is usually based on the patient’s clinical history. Hypotension due to generalized exposure to the cold is described both in the typical and the atypical forms.

**Case presentation:**

We describe a 9-year-old boy who, at the beginning of the summer after the first swim in the sea, developed generalized urticaria, dyspnea, conjunctival hyperemia, blurred vision and loss of strength. The child was treated with intramuscular steroid and intravenous antihistamine, and the symptoms quickly resolved. Insect bite, contact with fish and drug ingestion were denied, and no unusual food had been eaten before the swim. A tentative diagnosis was made of either aquagenic urticaria or cold urticaria, but the specific tests were negative. Although the cause was unknown, prophylactic treatment with antihistamines was prescribed but in spite of this, wheals developed all over the body, after every swim in the sea. The child then came to our attention and relying on clinical history a diagnosis of atypical cold urticaria was made: development of hives even in areas not directly exposed to cold and a negative response to the cold stimulation test, are the characteristic features of this rare form of cold urticaria.

**Conclusion:**

Atypical cold urticaria should be suspected in all cases of anaphylaxis related to cold exposure (i.e. contact with water) with a negative cold stimulation test.

## Background

Cold-induced urticaria (CU) is a physical urticaria characterized by the appearance of wheals after exposure to cold. It represents one third of all cases of physical urticaria [1]. The most well-known form of CU is the “typical” one (TCU), characterized by the appearance of wheals only in cold stimulated areas and confirmed by a positive response to the specific cold stimulation test (CST). In the “atypical” form (ACU) on the other hand, hives appear even in areas not directly exposed to the cold and the standard CST is negative [2] so its diagnosis relies largely on patient’s clinical history. ACU comprises about 20% of all acquired CU, both in adults and in children [3, 4], but data regarding this sub-type of CU are currently scarce and conflicting, especially in pediatric population. In both forms, hypotension due to generalized exposure to cold is described, but no recent case report of a systemic reaction misunderstood for food or contact anaphylaxis has been reported.

## Case presentation

**Fig. 1:**
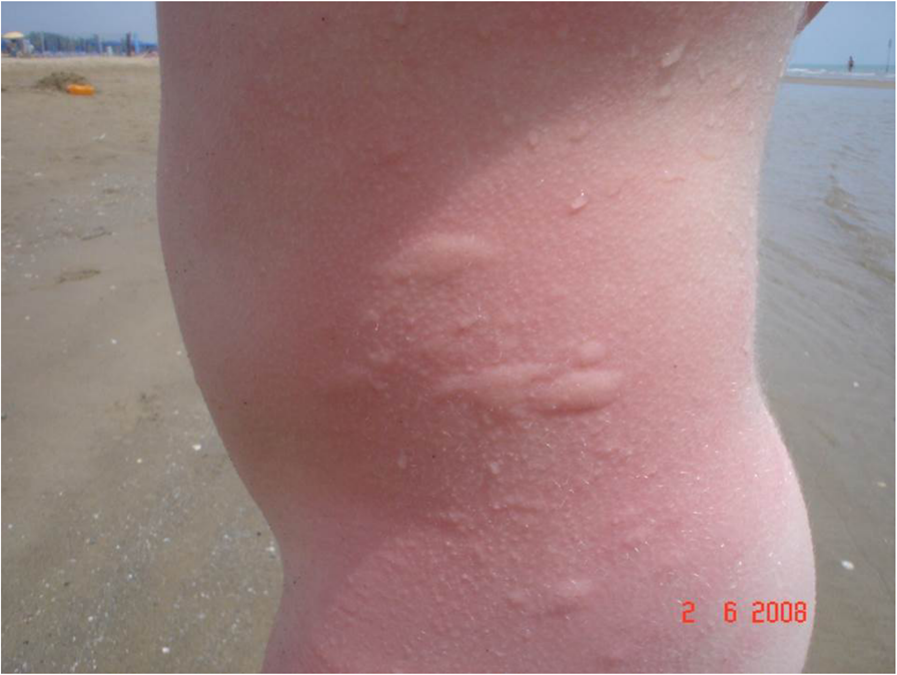
Wheals developed on the whole body of the patient after immersion in seawater

A 9-year-old boy complained of malaise, just a few minutes after his first summer swim in the sea; soon after, he presented generalized urticaria, dyspnea, conjunctival hyperemia, blurred vision and faintness. When first aid arrived, since anaphylactic shock was suspected, intramuscular steroids, intravenous antihistamine and nebulized salbutamol were administered, with rapid improvement on the part of the patient. The only thing of note in the child’s medical history was allergy to dust mite, and no other allergies were reported. There was no evidence of any insect bite or drug ingestion; an hour before the swim, the child had eaten his usual breakfast, with hot chocolate. Apparently, there was not contact with fish during the swim. The child had never complained of similar symptoms before and had never had urticaria after contact with water, be it seawater or tap water. No familiarity for allergic disease or chronic urticaria was reported. The child was referred to the local Allergy Department and in order to identify the offender, skin tests and specific IgE assays were performed. In detail, they tested allergy to milk, due the history of milk intake before the appearance of symptoms, and to insect venom and fish, because of the possibility of contact with insects and fish during the bath; all the tests were negative. Although the patient developed no symptoms on contact with tap water, an aquagenic urticaria was suspected, but the specific test was negative. Finally, a cold urticaria was suspected but the cold stimulation test (CST) was negative too. Given the severity of the reaction, prophylactic antihistamine therapy was commenced, but in spite of this, throughout the summer the patient continued to develop wheals all over his body after every swim in the sea (Fig. 1), even in places where there had been no direct contact between the skin and the water. The child then came to our attention, at the Burlo Garofalo Institute for Maternal and Child Health in Trieste (Italy), the referral centre for allergic diseases in the north-east of Italy. There, based on the child’s clinical history, a diagnosis of an atypical form of cold urticaria (ACU) was formulated. The specific diagnostic test of ACU involves keeping the lightly clothed patient in a cold room (at a temperature of 4 °C) for 30 min; in our case, it was avoided because of the past patient’s severe systemic reaction. In any case, the boy’s history was fairly characteristic enough to confirm the diagnosis of this rare and often unrecognised chronic physical urticaria: typical diagnostic features of ACU are in fact the appearance of wheals after exposure to various sources of cold (such as seawater at the beginning of summer), also in areas not directly in contact with water and the negativity of the CST. Antihistamine therapy was continued for the whole summer with fair control of symptoms and self-injectable epinephrine was prescribed but the child has never used it.

## Discussion

Urticaria is a common finding in pediatrics. It is characterized by the presence of wheals and\or angioedema. Wheals are skin lesions characterized by a central swelling and peripheral erythema; the single skin lesion lasts 1–24 h, but the duration of the whole disease can be longer [5]. Angioedema is characterized by sudden erythematous swelling of skin or mucous [5]. The most common form of urticaria in children is acute spontaneous urticaria, that often is triggered by unspecific viral infections; more rarely acute urticaria is correlated to food or drugs allergy. In these last cases, urticaria is usually associated to other allergic manifestations (i.e. rhinitis, conjunctivitis, bronchospasm, abdominal pain, etc.) and usually lasts few hours, while in the forms due to viral infections, urticaria usually lasts longer.

If urticaria lasts more than 6 weeks, it is defined as chronic urticaria. The prevalence of chronic urticaria in children is about 0.1–0.3% [6] and it has been found to be more common among atopic patients or patients with familiarity for autoimmune diseases [7]. In about 80% of cases, chronic urticaria is idiopathic, even if almost half of these patients presents autoantibodies, whose presence could be demonstrated by an autologous skin test [8]. Even if the sensibility and specificity of the autologous skin test has been demonstrated to be low [9], this minimal invasive technique is widely used because it can be easy and quickly performed and because it has an high negative predictive value for autoimmune CU [10]. The remaining patients present inducible forms of urticaria, that can be caused by many different stimuli: physical (such as cold urticaria, dermographic urticaria, delayed pressure urticaria, heat urticaria, solar urticaria, aquagenic urticaria, vibratory angioedema), or by exercise and hot bath (cholinergic urticaria) [5]. Physical urticarias account for 22% of chronic urticaria in children, and among these dermatographism is the most common [7]. Recently, European and US guidelines for the diagnosis and the management of CU have been published; the two guidelines are mostly similar even if some differences, especially about treatment, are still present [11].

As previous reported CU accounts for about 30% of physical urticaria in childhood, even if its prevalence may vary according latitude [12]. It is more common among young adults, but it can affect also young children [13]. The pathogenesis of CU is not well defined, but the presence of IgE autoantibodies that react against specific skin antigens only at low temperature has been hypothesized [13]. According to the result of CST, CU can be classified in two forms: the TCU (with a positive CST) and the ACU (with a negative test). The TCU can be primary or secondary to infections, neoplasms, cryoglobulinemia, vasculitis and drugs [14]; since secondary forms are very rare in pediatrics, specific tests should not be performed unless an evocative clinical history [5]. ACU can be classified in different subtypes according to the clinical presentation: the most common is systemic ACU. Other rarer forms are: localized ACU, cold-induced cholinergic urticaria (in which wheals develop after physical exercise but only if it is performed in a cold setting), cold dependent dermatographism (that develops only in areas exposed to cold), delayed cold urticaria (in which wheals develop, in cold stimulated areas, 12–48 h after stimulus aplication) and localized cold-reflex urticaria (that is characterized by appearance of wheals at 5-8 cm from the cold stimulated area)[14]. Finally, also autosomal dominant familial forms of CU have been described: the familial delayed cold urticaria and the cold-induced autoinflammatory syndrome. The last is a very rare cryopyrine-associated periodic syndrome and it is usually characterized by early onset, long lasting skin lesions and presence of other symptoms such as fever, arthritis, etc. [14].

The systemic form of ACU is rare, but it should be promptly recognized because it is usually characterized by more severe reactions then TCU. Unlikely, its diagnosis is often challenging because of the lack of specific diagnostic tests and because it can be precipitated by many different sources, kinds and degrees of cold. Moreover, the clinical association with the cold is usually less obvious than in TCU: in fact, in ACU, the appearance of wheals is caused by a decrease in body temperature rather than by direct contact with a cold object [15]. It is also well known that there are patients those only reacted to specific kinds of weather (increased humidity, for example) [16] and that unique environmental conditions could be sometimes been required to cause the development of wheals in ACU[2].

It is by now recognized that in TCU, generalized exposure to the cold (for example, falling into cold water) can cause severe systemic symptoms, including hypotension [14]; systemic anaphylaxis was recently described in a 9-year-old child with TCU [17].

A recent retrospective study [18] compared clinical characteristics of adult and pediatric patients with TCU and ACU: no difference was found about sex, median age at onset, but the percent of patients with a negative CST was higher among children than adults (50% vs 20.4%, *p* = 0.01). No statistically significant difference regarding symptom severity were found between ACU and TCU patients, but severe systemic reactions (dizziness, sensation o fainting, disorientation, shock, short of breath) were more common in the TCU group than in the ACU one (18.9% vs 4.8%, *p* = 0.14) and among children than adults (25% vs 16.7%, *p* = 0.70) [18]. Opposed results were found in a cohort of 30 children with CU: systemic reactions were present in 66.7% of children with negative CST and in 41.7% of children with positive CST (p = 0.14) [4]. No risk factor for systemic reactions was found [4].

There is no specific diagnostic test for ACU, although some Authors suggest a specific test in which the patient, wearing light clothes, is to be placed in a cold room (at a temperature of 4 °C) for 30 min [19]. This test is not commonly used because of the difficulties in carrying it out and the high risk of severe systemic reactions. In any case, the atypical form of CU is easy to recognize if its unmistakable characteristics are familiar.

Just like the typical form of CU, ACU usually responds well to antihistamine therapy, both in presence of acute symptoms and for prophylaxis [20]. According to Deza et al. [18], percent of patients achieving disease control using only antihistamines was similar between ACU and TCU patients; nevertheless, in the ACU the antihistamine dosage required was lower.

The other key aspect of treatment is prevention: patients should avoid abrupt exposure to intense cold and should take prophylactic antihistamine therapy if a high-risk activity is programmed (such as sailing in winter); in high-risk patients self-injectable epinephrine should also be prescribed [12, 13]. Even if there are no clear risk factors, it seems reasonable that patients who had only mild reactions after swimming could continue this activity with the previous described cautions and always under supervision by an adult trained in use of an epinephrine auto injector [4]. A recent survey by Gernez et al. found that the majority of allergy and immunology specialist infrequently prescribes epinephrine auto injector in patients with CU; the decision is usually based on past symptoms (especially laryngeal symptoms with cold beverages) and on patient participation in water-related or cold-related activities [21].

Even if life-threating symptoms, as the ones described in our patient, are possible, most patients with CU have mild and intermittent symptoms. The mean duration of symptoms of CU is 4.8 to 7.9 years [1]; disease duration in ACU seems to be shorter than in the TCU (4.3 vs 5.3 years, *p* = 0.02) [18].

## Conclusion

ACU can show with severe systemic symptoms until anaphylaxis, so it must be suspected in all cases of anaphylaxis related to any kind of generalized cold exposure and in all cases of CU related to cold exposure, especially in those children who seem to be more likely to have generalized symptoms. The case described is emblematic of the potential severity of this condition and shows the challenges involved in recognizing this rare form of urticaria. However, diagnosis can be easily made without the need for allergy tests just by remembering the typical features of ACU: 1) correlation to various cold stimuli, 2) appearance of wheals even in areas not directly exposed to the cold and 3) a negative response to the cold stimulation CST.

## Abbreviations

ACU: Atypical cold-induced urticaria
CST: Cold stimulation test
CU: Cold-induced urticaria
TCU: Typical cold-induced urticaria
